# The Association Between Sleep Disturbance and Suicidality in Psychiatric Inpatients Transitioning to the Community: Protocol for an Ecological Momentary Assessment Study

**DOI:** 10.2196/33817

**Published:** 2022-05-17

**Authors:** Lindsay H Dewa, Sofia Pappa, Talya Greene, James Cooke, Lizzie Mitchell, Molly Hadley, Martina Di Simplicio, Thomas Woodcock, Paul Aylin

**Affiliations:** 1 School of Public Health Imperial College London London United Kingdom; 2 National Institute for Health and Care Research Imperial Patient Safety Translational Research Centre Imperial College London London United Kingdom; 3 West London National Health Service Trust London United Kingdom; 4 Department of Brain Sciences Imperial College London London United Kingdom; 5 Department of Community Mental Health University of Haifa Haifa Israel; 6 Division of Psychiatry University College London London United Kingdom; 7 Applied Research Collaboration Northwest London Imperial College London London United Kingdom

**Keywords:** sleep, suicide, psychiatric inpatient, ecological momentary assessment, EMA, experience sampling, coproduction, sleep disturbance, discharge

## Abstract

**Background:**

Patients are at high risk of suicidal behavior and death by suicide immediately following discharge from inpatient psychiatric hospitals. Furthermore, there is a high prevalence of sleep problems in inpatient settings, which is associated with worse outcomes following hospitalization. However, it is unknown whether poor sleep is associated with suicidality following initial hospital discharge.

**Objective:**

Our study objective is to describe a protocol for an ecological momentary assessment (EMA) study that aims to examine the relationship between sleep and suicidality in discharged patients.

**Methods:**

Our study will use an EMA design based on a wearable device to examine the sleep-suicide relationship during the transition from acute inpatient care to the community. Prospectively discharged inpatients 18 to 35 years old with mental disorders (N=50) will be assessed for eligibility and recruited across 2 sites. Data on suicidal ideation, behavior, and imagery; nonsuicidal self-harm and imagery; defeat, entrapment, and hopelessness; affect; and sleep will be collected on the Pro-Diary V wrist-worn electronic watch for up to 14 days. Objective sleep and daytime activity will be measured using the inbuilt MotionWare software. Questionnaires will be administered face-to-face at baseline and follow up, and data will also be collected on the acceptability and feasibility of using the Pro-Diary V watch to monitor the transition following discharge. The study has been, and will continue to be, coproduced with young people with experience of being in an inpatient setting and suicidality.

**Results:**

South Birmingham Research Ethics Committee (21/WM/0128) approved the study on June 28, 2021. We expect to see a relationship between poor sleep and postdischarge suicidality. Results will be available in 2022.

**Conclusions:**

This protocol describes the first coproduced EMA study to examine the relationship between sleep and suicidality and to apply the integrated motivational volitional model in young patients transitioning from a psychiatric hospital to the community. We expect our findings will inform coproduction in suicidology research and clarify the role of digital monitoring of suicidality and sleep before and after initial hospital discharge.

**International Registered Report Identifier (IRRID):**

PRR1-10.2196/33817

## Introduction

### Suicidal Ideation and Behavior After Psychiatric Hospital Discharge

The prevention of suicide is a well-established goal for patient safety and a national and global public health priority. Suicide risk is increased after discharge from a psychiatric hospital [1], regardless of previous suicidal behavior. In the United Kingdom, there were 1988 deaths by suicide between 2008 and 2018 within 3 months of discharge from inpatient care, which amounts to 15% of all suicides [2]. Suicide is most frequent in the first week following discharge, making the transition period from hospital to the community one of the riskiest for suicidal behavior in adults and young people. In fact, this risk is highest on day 2, yet follow up with support services sometimes does not occur until day 7 [3]. Thus, the first week is a critical period for immediate aftercare and treatment planning following discharge from psychiatric inpatient care. The risk is significantly increased in patients with various individual factors, including previous self-inflicted violence (eg, self-harm and suicidal behavior), intoxication, and health conditions. Risk is further elevated when patients experience insomnia, nightmares, or sleep disturbance that results in being awake at night [4]. This is particularly troublesome as immediate support (eg, clinical services or social support networks) is typically limited during the night.

### The Relationship Between Sleep, Suicidal Ideation, and Behavior

There is increasing evidence that sleep dysfunction is an independent risk factor for suicidal ideation and behavior (SIB) [5]. Several systematic reviews have explored the epidemiological relationship between sleep and SIB in different population groups (eg, in mixed samples [6], nonpsychiatric adolescent patients [7,8], and psychiatric patients [9]), examined prospective suicide incidents, and more recently, explored the underlying mechanisms of this relationship [9]. All these studies have showed a strong correlation between sleep and SIB; however, most studies have been cross-sectional or retrospective in design. The temporal nature of the relationship between sleep disturbance and suicidality was the focus of the most recent systematic review [10]. Of the 41 included studies, only 1 measured insomnia as a predictor of suicidal ideation in real time (over a 24-hour period) in the United Kingdom [11]. The lack of studies may be due to the challenge and expense of monitoring sleep and suicidality without using self-reported data. However, the scarcity of data is problematic, since both sleep and suicidality fluctuate; therefore, current studies do not account for temporality [12-15].

### Measuring Sleep and SIB in Real Time Using Technology

Ecological momentary assessment (EMA) is an intensive self-report method that can be used to assess experiences, symptoms and behaviors in real-time at multiple times of the day [16,17]. EMA monitors people within their natural environment, usually with the aid of a smartphone or wearable smart device (such as a wrist-worn electronic watch) to reduce recall bias, leading to greater efficiency, ecological validity, and accuracy than more traditional research methods [18]. The use of EMA in the study of SIB has shown great promise because of its ability to monitor and identify proximal factors (eg, sleep) associated with SIB in daily routines; the use of EMA has increased dramatically in the last 5 years [19,20]. However, only a few EMA studies have been conducted in high-risk groups with experience of suicidal ideation and behavior during inpatient hospitalization or following discharge [12,21-24]. Most recently, Glenn and colleagues [22,25] performed an EMA study with a 28-day period to examine the relationship between sleep and suicidality in adolescents (12-18 years old) following discharge from acute psychiatric care in the United States. Notably, EMA was deemed feasible in this high-risk group, with adherence being highest in the first week following discharge; participants found that wearing an actigraphy device (eg, a wearable device to measure the sleep-wake cycle) was acceptable. Similarly, EMA has also been deemed feasible to measure SIB in patients undergoing community mental health support [25] and acceptable as a data collection method in young adult cohorts [25,26]. However, to our knowledge, no studies have used EMA to monitor sleep and suicidality during the transition from psychiatric inpatient care to the community.

### Application of Sleep to the Integrated Motivational Volitional Model

While there is increasing evidence of a prospective relationship between sleep problems and suicide, not everyone who experiences sleep problems will develop suicidal thoughts and behavior, or vice versa. Therefore, it is vital to investigate the underlying mechanisms by which disturbed sleep may lead to suicidality. The integrated motivational volitional (IMV) model is an established theoretical model that proposes that people transition from ideation to suicidal behavior because of the complex interplay of multiple factors, including core psychological mechanisms, such as defeat and entrapment, and background factors (eg, life events such as being in hospital) [27,28]. Littlewood and colleagues [29] recognised the potential importance of suicidal imagery (ie, “flashforwards” of future events involving one’s suicide or the aftermath of death) when studying the relationship between sleep and suicide. Moreover, suicidal imagery might also trigger entrapment or hopelessness, resulting in suicidal ideation and behavior [28,29]. These conceptualizations are promising, but to date have not been examined in young adults transitioning from the hospital to the community. This proposal therefore presents a unique opportunity to address these gaps in research and patient safety in this vulnerable population.

The primary aim of this paper is to describe and outline an exploratory EMA study protocol that examines sleep as a possible measure of deterioration and a potential predictive factor for SIB in young patients during the transition period from an inpatient setting to the community. We will address the following research questions: (1) Is there a difference between predischarge and postdischarge sleep in young adult patients? (2) Does sleep disturbance pre- and postdischarge predict postdischarge SIB in young patients? and (3) Is it acceptable and feasible to use EMA to monitor SIB postdischarge in young adults?

We hypothesize that there will be a difference between pre- and postdischarge sleep in young patients (this is hypothesis 1). Additionally, based on previous research, we hypothesize that sleep disturbance (ie, insomnia and short sleep duration) postdischarge will predict SIB while controlling for depression and baseline sleep (this is hypothesis 2). We will also explore the conceptual model in our patient sample; we expect that sleep disturbance will be associated with defeat, entrapment, and hopelessness postdischarge (this is hypothesis 3). We also expect that EMA will be deemed both feasible and acceptable for inpatients transitioning from the inpatient setting to the community.

## Methods

### Study Design and Setting

**Figure 1:**
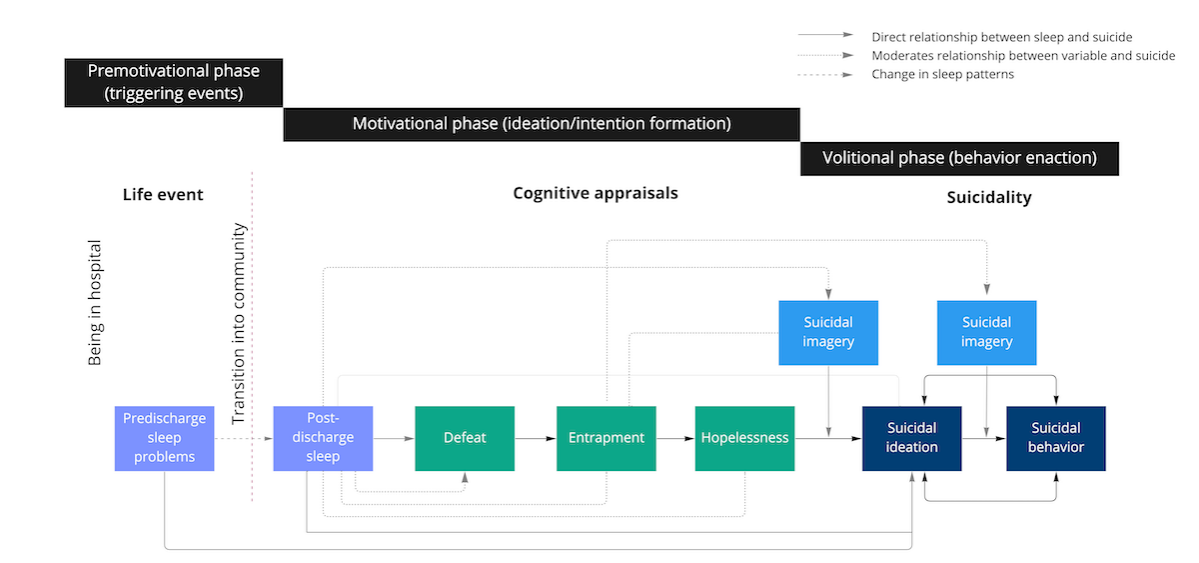
Adapted conceptual model of sleep and suicide in young transitioning inpatients.

This is a prospective, repeated-measures EMA study with inbuilt evaluation. Wrist-worn digital devices will be used to explore the relationship between sleep and SIB during an acute psychiatric transition while also evaluating defeat, entrapment, hopelessness, and suicidal imagery. Our conceptual model integrates key aspects of the IMV model [27,28] and is one of only two psychological models to integrate sleep [29] (Figure 1).

Recruitment will take place in 2 acute psychiatric hospitals in West London that provide support and care for mental health problems or mental health crises (Hammersmith and Fulham Mental Health Unit and Lakeside Mental Health Unit). EMA using smartphones is a practical option; however, it is difficult to accurately measure the sleep-wake cycle with this method [30] and there are potential privacy issues [31] in retrieving data due to connectivity; moreover, some patients might not have access to a smartphone within an acute hospital environment in England. Hence, we opted for the Pro-Diary V device (Camntech), which has inbuilt actimetry, and short questionnaires as the most viable options for accuracy and ease of use within the flow of daily life.

### Ethics Approval

This study received ethical approval from South Birmingham Research Ethics Committee (21/WM/0128) on June 28, 2021. The study will be registered on the Observational Studies Register.

### Eligibility

Participants will be inpatients in an acute psychiatric ward due to be discharged within a 4-week period, age 18 to 35 years old, with a diagnosis of a mental disorder confirmed by the Structured Clinical Interview from the Diagnostic and Statistical Manual of Mental Disorders, Fifth Edition, in their clinical records [18]. Participants will be excluded if they are outside the age range, not fluent in English, or using sleep medication (ie, hypnotic medication such as zopiclone or zolpidem). We will not exclude participants on anxiolytics (ie, benzodiazepines) as most patients discharged will likely be on medication with some sedative effect.

### Recruitment

Clinicians at West London National Health Service (NHS) Trust (WLT) will initially approach eligible participants in the acute psychiatric hospitals participating in the study. Prospective participants will be given an information sheet and asked if they would be happy for a researcher to approach them. If they agree, a trained researcher will approach them, explain the study, and answer any questions. After at least 24 hours, the patient will be approached for a second time to ask if they would be happy to take part in the study; consent will then be obtained.

### Procedure

Following screening and informed consent, key demographic factors will be collected via medical records. These will include gender, age, ethnicity, marital status, length of time in hospital, mental health diagnosis, prescribed medication (historic and current), sleep history, family history of suicide, previous suicidal thoughts, and suicide plans and attempts. The researcher will then administer the baseline questionnaires face-to-face at a date and time convenient to the participant.

The researchers will then brief each participant during a 15-minute one-on-one session on the purpose of the Pro-Diary V watch, procedures needed to complete the study, and the method of using the watch, and will give accompanying written guidance on how to use the wearable device (eg, not to take the watch off unless having a shower or bath, how to use the menus, and how to answer the questions). The researchers will show the participants a demonstration version of the Pro-Diary V watch (demo beeps will be enabled for user training), and will prompt the participants with a timed questionnaire to allow the participants to learn the operation of the device. The participants will not need to charge the watch, as it has a battery life longer than the duration of the data collection period. The researchers will go through the written guidance with the participants to make sure they have a good understanding of what is expected of them and will answer any questions they might have. Selected onsite staff at each inpatient unit will be trained in the use of the watch and will assist the participants daily until discharge.

**Figure 2:**
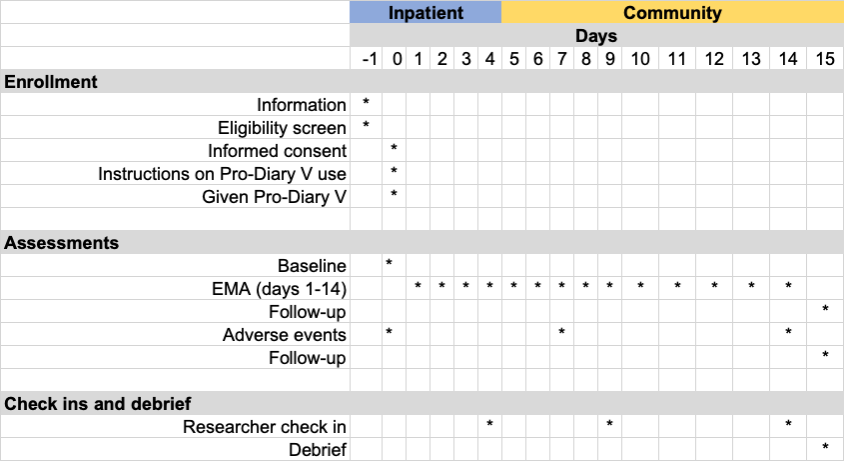
Study schedule. EMA: ecological momentary assessment.

**Figure 3:**
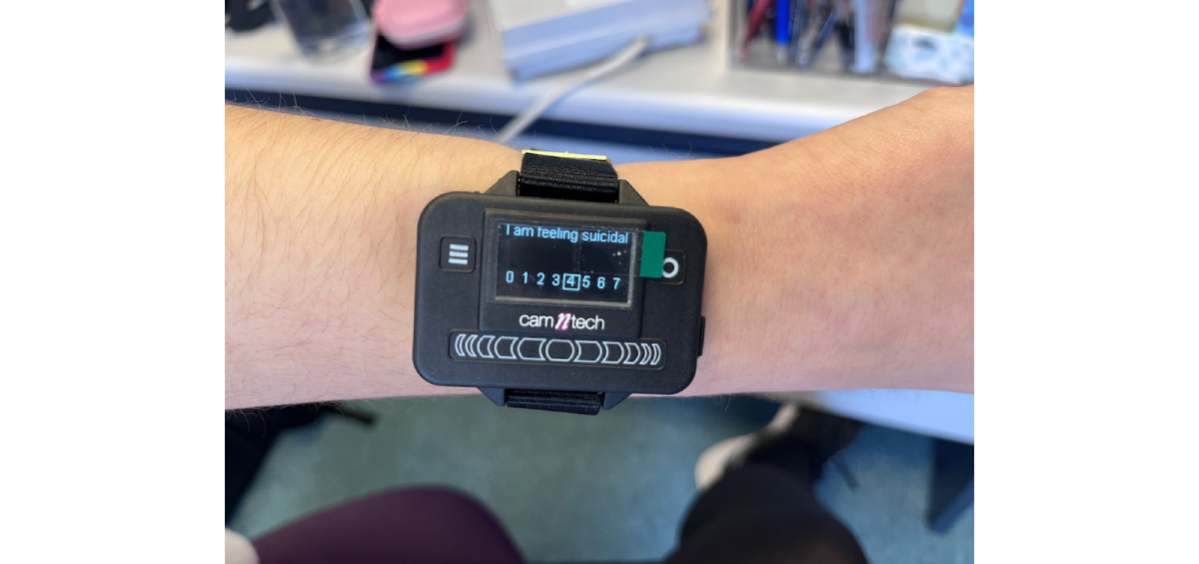
Example of Pro-Diary V watch with question about suicidal ideation.

The participants will wear the watch for a maximum of 14 days, including 1 to 4 days before discharge, to determine the baseline profile and a minimum of 10 days after discharge (ie, the riskiest period for SIB and death by suicide). During this period, the watch will prompt each participant 4 times per day by vibrating to answer brief questions (Figure 2, Figure 3). The start point of the daily prompts will be decided by each participant based on their sleep-wake schedule. The start point of the day will normally be 30 minutes after their usual waking time. Between-prompt intervals will be delivered using a fixed time sampling schedule. Fixed time points were chosen to reduce study burden in this high-risk group of participants and increase compliance. Participants will be able to delay the prompt for 20 minutes if they are not able to answer straight away (eg, if they are undergoing inpatient activity). Each participant will receive a £15 (US $18.78) e-voucher after each stage (ie, they will receive a voucher after baseline assessment, after EMA, and after follow-up assessment), following a suggestion by our young coresearchers.

After discharge, the participants will receive 2 follow-up calls (once a week) to resolve any problems arising from the EMA method and the use of the watch, including technical issues, and to answer any queries from the participants, following existing guidance [32]. The researchers will contact the participants again toward the end of the EMA period to arrange a date to administer the follow-up questionnaires. The researchers will administer the questionnaires face-to-face in a private room at a WLT community site at a time convenient to the participant. At the end of the study period, deidentified EMA data will be collected from each participant, downloaded to a secure enclave at Imperial College London, and analyzed within the secure platform.

### Patient and Public Involvement

This study will build on our extensive experience of working with young people with mental health difficulties [26,33,34] to conduct meaningful patient and public involvement. We issued a call for young people with experience of psychiatric inpatient care and suicidality (eg, ideation or behavior) in November 2020 through existing advisory groups, email distribution lists, and social media (eg, Twitter) and asked prospective participants to fill out a short application form. After internal selection, 3 participants were selected to inform most research stages, serving as coresearchers. These coresearchers were consulted on (1) design and ethics, (2) recruitment, (3) management, (4) data interpretation, (5) dissemination of findings, and (6) evaluation. The coresearchers have already reviewed the research documentation (eg, the information sheet, protocol, and consent form) and subsequently, changes were made to the documents. For example, the language was revised into plain English and the “nightmares” variable was added to the design. A recruitment poster was also coproduced with the coresearchers. One coresearcher also attended the NHS Research Ethics Committee meeting. The coresearchers have been trained in a basic understanding of EMA and sleep. Based on a suggestion from a previous coresearcher, [26] we expect to cofacilitate a face-to-face “meet and greet” event at the acute hospital for the research team to meet the potential study participants and encourage participation. We have set up a project-specific WhatsApp (Facebook Inc) group to make sure everyone is easily involved in decision-making, and we will have 4 virtual team meetings per year on Zoom (Zoom Inc). Coresearchers will also be listed as coauthors on all study outputs, including academic papers. Disclosure and Barring Service certificates have been obtained or are currently being obtained for coresearchers to ensure that all are deemed capable of safely interacting with patients with mental health difficulties.

### Baseline and Follow-up Questionnaires

Participants will complete a baseline assessment (Table 1) that will cover sleep with the Sleep Condition Indicator (SCI) and Pittsburgh Sleep Quality Index (PSQI) [35,36], nightmares with the Disturbing Dream and Nightmare Severity Index [37], sleep environment with the Hospital Environment Sleep Questionnaire [38], affect with the Positive and Negative Affect Scale [39], depression with the Patient Health Questionnaire (PHQ-9) [40], anxiety with Generalized Anxiety Disorder-7 scale [41], defeat and entrapment with the Short Defeat and Entrapment Scale [42]), mental imagery with the Impact of Future Events Scale [43], and lifetime suicidal and nonsuicidal ideation and behavior with the Self-injurious Thoughts and Behavior Interview [44]. Additional follow-up questions will cover medication use, access to hospital and primary care, and informal or formal support received since discharge. Questions on the acceptability of using EMA and a wearable device to monitor the transition were adapted from another study that used EMA (questions included, for example, “I found the questions easy to understand” [45]) or were included after discussion with the study authors and our wider team. Feasibility will be measured by participation rate, adherence to the EMA protocol, and adherence to wearing the device.

**Table 1:** Baseline and follow-up measures.

Variable	Questionnaire	Items	Scoring	Used at baseline, follow up, or both
Sleep environment	Hospital Environment Sleep Questionnaire	16	0-64	Baseline
Sleep	Sleep Condition Indicator	8	0-32	Both
Sleep	Pittsburgh Sleep Quality Index	19	0-21	Both
Nightmares	Disturbing Dream and Nightmare Severity Index	6	Unique to item	Both
Affect	Positive and Negative Affect Scale	20	10-50	Both
Depression	Patient Health Questionnaire-9	9	0-27	Both
Anxiety	Generalized Anxiety Disorder-7 scale	7	0-21	Both
Mental imagery	Impact of Future Events Scale	24	0-96	Both
Defeat and entrapment	Short Defeat and Entrapment Scale	8	0-16 for defeat and 0-16 for entrapment	Both
Lifetime and current suicidal and nonsuicidal self-injury ideation and behavior	Self-injurious Thoughts and Behavior Interview-Short Form	N/A^a^	Unique to item, including open ended, counts, and 0-4 scale	Baseline (lifetime) and follow up (current only)
Feasibility and acceptability	Bespoke questionnaire designed to measure feasibility and acceptability of ecological momentary assessment for mental health following discharge	10	1-50 and open ended	Follow up

^a^N/A: not applicable.

### EMA Measures

EMA items were selected according to use in previous studies [46], adapted from standardized measures, or taken from the experience sampling methodology (ESM) item repository [47] (Table 2 and Table 3). Some were adapted after discussion with the young coresearchers. For example, “Right now, I am feeling suicidal” was revised to “Right now, I feel suicidal,” as it was deemed simpler to read and more in line with the structure of the other items. Similarly, questionnaire density, sampling scheme, and momentary assessment frequency were informed by the considerations of the coresearchers, by the characteristics of the high-risk population, and by previous literature. Previous literature shows that participants in studies with a shorter duration and a lower number of question prompts per day had higher compliance [48]. Therefore, we considered that minimizing the number of prompts per day and limiting the study period to 14 days would result in the highest participant compliance rate [21]. We discussed this idea with the coresearchers and decided that 4 was a reasonable number of prompts. This was based on consideration of the high-risk population and an opinion that 4 prompts did not impose an undue burden and would still provide enough data to capture different times of the day.

**Table 2:** Ecological momentary assessment measures. Measures were taken 4 times daily and used a 7-point Likert scale, ranging from 1 (“not at all”) to 7 (“very much so”).

Variable	Item
Suicidal ideation [11]	Right now, I feel suicidal
Self-harm ideation [46]	Right now, I feel like harming myself without the intention to die
Defeat [42]	Right now, I feel defeated by life Right now, I feel powerless
Entrapment [42]	Right now, I feel trapped Right now, I want to escape my emotional pain
Positive affect^a^	Right now, I feel excited Right now, I feel cheerful Right now, I feel satisfied Right now, I feel relaxed
Negative affect^a^	Right now, I feel stressed Right now, I feel irritated Right now, I feel anxious Right now, I feel sad Right now, I feel insecure Right now, I feel hopeless

^a^Taken from the experience sampling methodology (ESM) item repository [47].

**Table 3:** Daily measures.

Variable	Item	Frequency	Response scale	
Suicidal behavior	I tried to kill myself today	Last beep of the day	Binary^a^	
Self-harm	I have self-harmed today	Last beep of the day	Binary	
Suicidal imagery	I have had images of making a suicide attempt today	Last beep of the day	Binary	
Self-harm imagery	I have had images of hurting myself today	Last beep of the day	Binary	
Nightmares [49]	Did you have nightmares of a traumatic experience last night?	First beep of the day	Binary	
Subjective sleep parameters [50]	What time did you get into bed?	First beep of the day	Time	
	What time did you try and to go to sleep?	First beep of the day	Time	
	How long did it take you to fall asleep?	First beep of the day	Minutes	
	How many times did you wake up, not counting your final awakening? In total, how long did these awakenings last?	First beep of the day	Number	
	What time was your final awakening?	First beep of the day	Time	
	What time did you get out of bed for the day?	First beep of the day	Time	
	How would you rate the quality of your sleep?	First beep of the day	Likert^b^	

^a^In binary scales, 0 indicated “no” and 1 indicated “yes.”

^b^Five-point Likert scale, ranging from 1 (“very poor”) to 5 (“very good”).

### Objective and Subjective Sleep Parameters

A total of 5 key parameters will be objectively measured via inbuilt actimetry in the Pro-Diary V watch: total sleep time, sleep efficiency, wake after onset, sleep latency, and the number of awakenings. The same parameters will be measured through self-reporting using the Pro-Diary V software sleep diary, based on the Consensus Sleep Diary [50], to validate the objective reporting. This is recommended practice to establish the key period for analyzing sleep parameters [51].

### Statistical Analysis

The lme4 and nlme R CRAN packages (CRAN R Project) for mixed effects models will be used. We will measure subjective sleep and objective sleep (via actigraphy) separately; this will be accounted for in independent models. Suicidal ideation will be measured multiple times a day. We will calculate the next-day worst point levels (eg, the highest score each day) for the suicide parameters [22] (eg, postdischarge sleep and early morning suicidal ideation), as this is deemed to be the best predictor of suicidal behavior [52]. In the first instance, analysis will be conducted to answer the primary research questions and hypotheses with specified confounders. A secondary analysis will be conducted for more exploratory work and to determine the conceptual model relationships between subjects and data. To address hypothesis 1, we will analyze both person-level baseline sleep problems (determined by the SCI and PSQI) and within-person sleep diary predictors (determined by EMA and actigraphy). We will first compare baseline sleep problems for mean values and follow-up mean values with the *t* test. Next, we will conduct a linear regression analysis to examine the grand-mean centered daily level (within-person) sleep diary variables from the night before (total sleep time, sleep efficiency, wake after onset, sleep quality, sleep latency, the number of awakenings, and nightmares) in the inpatient hospital (days 1-4) and post-discharge sleep parameters by data number (ie, time-related variables). A similar approach will be taken with the actigraphy data. To address hypothesis 2, all models will have random intercepts and use either postdischarge worst-point suicidal ideation across any day (days 5-14) or day-level suicidal behavior. Our first mixed effects model will include EMA (day level) sleep parameter predictors: total sleep time, sleep efficiency, wake after onset, sleep quality, sleep latency, the number of awakenings, and “nightmares.” Our second mixed effects model will add person-level variables, including baseline sleep (SCI and PSQI) and depression (PHQ-9). Our final model will add in the interactions between ESM day-level parameters and person-level variables that are significant (*P*<.05). The same models will be applied for the secondary outcomes (nonsuicidal self-injury ideation and behavior), and objective sleep parameters (the actigraphy data). We will then explore the conceptual model and specifically address hypothesis 3. We will use separate mixed effects models to examine the impact of EMA (momentary) entrapment, defeat, and hopelessness on awakening suicidal ideation (the start of day prompt) while moderating for each day-level sleep parameter predictor (EMA and actigraphy).

### Sample Size Calculation

Sample size calculations for EMA studies differ from traditional calculations due to the inherent multi-level structure of the design, with multiple assessment periods. However, to answer our primary research question (ie, “Does sleep disturbance pre- and postdischarge predict postdischarge SIB in young patients?), we need to calculate our sample size based on 3 levels of data: momentary assessments nested within days, nested within persons. Specifically, these are day-level observations of suicide (the worst point level for suicidal ideation among 4 momentary assessments) and sleep. In the absence of pilot data for this specific population, we estimated sample size based on several factors. First, the only 2 (to our knowledge) ESM studies [11,22] that examined sleep and suicide with similar clinical populations had roughly 50 participants (N=48 and N=51, respectively). Second, from this, we noted that a sample size of 50 was recognized as an acceptable sample size number for multi-level analyses, in line with guidance and simulation studies [53]. Third, we entered a sample size of 50 into an EMA calculator [54] to estimate the power with 14 days and 1 response (the worst point level of suicidal ideation) per day. This showed that a total of 50 participants was sufficient to achieve 90% power and detect large effect sizes, allowing for a 75% completion rate. To guard against possible dropout or incomplete EMA completion, we will dedicate time to the initial EMA briefing session with the participants and regularly check in with them to help maximize retention, in line with previous guidance [55] Notably, to our knowledge, few ESM studies have given justifications for sample size; therefore, we have tried to be as transparent as possible about our calculations. We will also extend the recruitment period if required. If there are missing data, we will apply maximum likelihood estimation to allow all data to be testable across the multilevel modeling.

### Ethical Approval, Considerations and Safeguarding

Ethical approval was obtained from NHS Ethics Committee and Health Research Authority (21/WM/0128). Upon discharge, as part of standard care, participants will receive a leaflet containing key contact details for their community support team and an additional booklet containing key numbers for each relevant informal support service (eg, Shout and Samaritans). These were co-designed with young people with lived experience. Participants will also be contacted by a member of the community mental health team within a week of discharge.

All participants involved in the study will be assessed for suicidal ideation and behavior at each EMA assessment using brief questions. After each assessment, a message will be displayed on the Pro-Diary V watch giving them contact information for their mental health services. This will be either their clinical team or the 24-hour single point of access (telephone number 0300 1234 244 in the United Kingdom); they will also be advised to go to the emergency department at their nearest hospital or call Shout—a 24-hour, 7-day-a-week crisis text line (available at 85258 in the United Kingdom) that aims to bring texters from a “hot moment” to “cool calm” through active listening and collaborative problem solving. Live, trained crisis counsellors receive text messages and immediately respond from a secure online platform. Participants will also be called by the researcher every week for a study check in.

Throughout the study, participants may be become incapacitated or rehospitalized and will therefore will be unable to continue. If this happens, we will contact the participant, if we are able to, and collect the wearable device. If we cannot contact the participant, we will ask the study inpatient clinician to collect it for us. We will explain that the data collected up until the point of being rehospitalized will be included in the data analysis, in line with the consent form and information sheet declaration.

## Results

Study recruitment was due to start in November 2021, and we expect results to be available in 2022. To date, we have reflected on the working relationship with the young coresearchers across each research stage. Researcher and coresearcher reflections indicate that establishing and maintaining a safe environment for open discussion and continued communication (eg, via a WhatsApp group) have been vital to effectively share power and decision-making. Safeguarding and support needs for both coresearchers (eg, an individualized strategy) and researchers (eg, clinical supervision) have also been particularly evident. To date, the coproduced design, recruitment poster, documentation (eg, the information sheet, protocol, and consent form), and this research paper have demonstrated significant impact.

## Discussion

EMA is a promising methodology that could monitor and identify factors (eg, sleep) associated with suicidality. In our qualitative study, we found that wearables were deemed acceptable and feasible by young patients to monitor sleep and activity and to detect mental deterioration [26]. However, it is unknown if wearables can be used in the real world to measure sleep and activity. This study is a unique opportunity to address these gaps in research and patient safety in this vulnerable population. We expect to find a relationship between poor sleep and next-day suicidal ideation. If successful, we expect to build on this work and examine this relationship, determining mechanisms with the adapted IMV model in a larger study sample. We also expect to implement and test sleep treatment for transitioning high-risk young patients to reduce suicidality.

There are potential key limitations to our study. First, our study sample is small. While adequately powered for our within-sample analyses, it will be less powered for the between-subjects analyses. Second, most momentary analyses will be exploratory, and we therefore will not be able to give a precise impression of the application of the adapted IMV model to sleep. However, we expect to develop this work in the future and conduct more highly powered studies. Lastly, unlike the gold standard measure of polysomnography, actigraphy cannot characterize a full sleep architecture. However, it does provide an objective measurement of sleep that is obtained within a natural environment, increasing ecological validity.

## Abbreviations

EMA: ecological momentary assessment
ESM: Experience Sampling Methodology
IMV: integrated motivational volitional
NHS: National Health Service
PHQ-9: Patient Health Questionnaire
PSQI: Pittsburgh Sleep Quality Index
SCI: Sleep Condition Indicator
SIB: suicidal ideation and behavior
WLT: West London National Health Service Trust
